# 3D Printed Auxetic Mechanical Metamaterial with Chiral Cells and Re-entrant Cores

**DOI:** 10.1038/s41598-018-20795-2

**Published:** 2018-02-05

**Authors:** Yunyao Jiang, Yaning Li

**Affiliations:** Department of Mechanical Engineering, University of New Hampshire, Durham, NH03824 USA

## Abstract

By combining the two basic deformation mechanisms for auxetic open-cell metamaterials, re-entrant angle and chirality, new hybrid chiral mechanical metamaterials are designed and fabricated via a multi-material 3D printer. Results from mechanical experiments on the 3D printed prototypes and systematic Finite Element (FE) simulations show that the new designs can achieve subsequential cell-opening mechanism under a very large range of overall strains (2.91%–52.6%). Also, the effective stiffness, the Poisson’s ratio and the cell-opening rate of the new designs can be tuned in a wide range by tailoring the two independent geometric parameters: the cell size ratio ${{\boldsymbol{c}}}_{{\bf{0}}}{\boldsymbol{/}}{{\boldsymbol{b}}}_{{\bf{0}}}$, and re-entrant angle *θ*. As an example application, a sequential particle release mechanism of the new designs was also systematically explored. This mechanism has potential application in drug delivery. The present new design concepts can be used to develop new multi-functional smart composites, sensors and/or actuators which are responsive to external load and/or environmental conditions.

## Introduction

Mechanical metamaterials are a category of lightweight artificial materials which can achieve unique mechanical properties via innovative geometric design^1–8^. The fast development of 3D printing enables rapid prototyping on arbitrary complex geometry and therefore provides a great opportunity to invent new mechanical metamaterials^9–14^. Within the family of mechanical metamaterials, auxetic mechanical metamaterials are one of the most interesting sub-families due to the effects of negative Poisson’s ratio^15–21^, and therefore the increased indentation resistance, shear resistance, superior energy absorption capability, and acoustic properties^22–35^. The auxetic mechanical metamaterials have broad engineering applications in designing energy absorption foams, smart composites, sensors and actuators, and biomedical devices and materials^9,36–39^.

For auxetic mechanical metamaterials, the negative Poisson’s ratio can be tuned by tailoring the geometry of each unit cell. The existing auxetic mechanical metamaterials, especially those with open-cell structures, follow two basic deformation mechanisms: symmetric units with re-entrant angles^22,40–42^, and chiral units that rotate when deformed^10,11,43–50^. Each mechanism has its own advantages. For the concept of re-entrant angle, dramatic auxetic effects can be effectively achieved and therefore the materials with re-entrant angle can easily reach the limiting Poisson’s ratio of −1 for isotropic material^22,40,41^, and by tailoring the anisotropy, the Poisson’s ratio can be further significantly reduced in one direction^51^. However, the auxetic mechanical metamaterials based on this mechanism can not sustain large compressive deformation, because the structure will lose stability and then lose auxeticity due to the breakage of re-entrant symmetry after instability^51^. Compared with this mechanism, auxetic mechanical metamaterials with chiral geometry have deterministic handedness and are expected to have more robust Poisson’s ratio performance under manufacturing errors with both small and large deformation^52–54^, although for quasi-isotropic open-cell auxetic chiral mechanical metamaterials, the Poisson’s ratio can not reach the lower limit of −1. While this drawback can be overcome with multi-material design or structure modification^55^.

Recently, it was found that the auxetic effect is positively correlated to the chirality-induced rotation efficiency, and by adding soft hinges or harder cores, the auxetic effects of chiral metamaterial can be significantly amplified^55^. In addition, by introducing a chiral core cell, a unique sequential cell opening mechanism can be achieved^56^. In this investigation, by fully utilizing the advantages of the two basic deformation mechanisms of re-entrant angle and chirality, new auxetic mechanical metamaterials are developed: a re-entrant core cell is introduced to the center of a missing-rib type of chiral cell. Therefore, a new hybrid cell with both design elements is generated. The new hybrid design shows new mechanical properties and wide tunability under a very large range of overall strain.

## Results

### Conceptual design and prototyping via 3D printing

**Figure 1:**
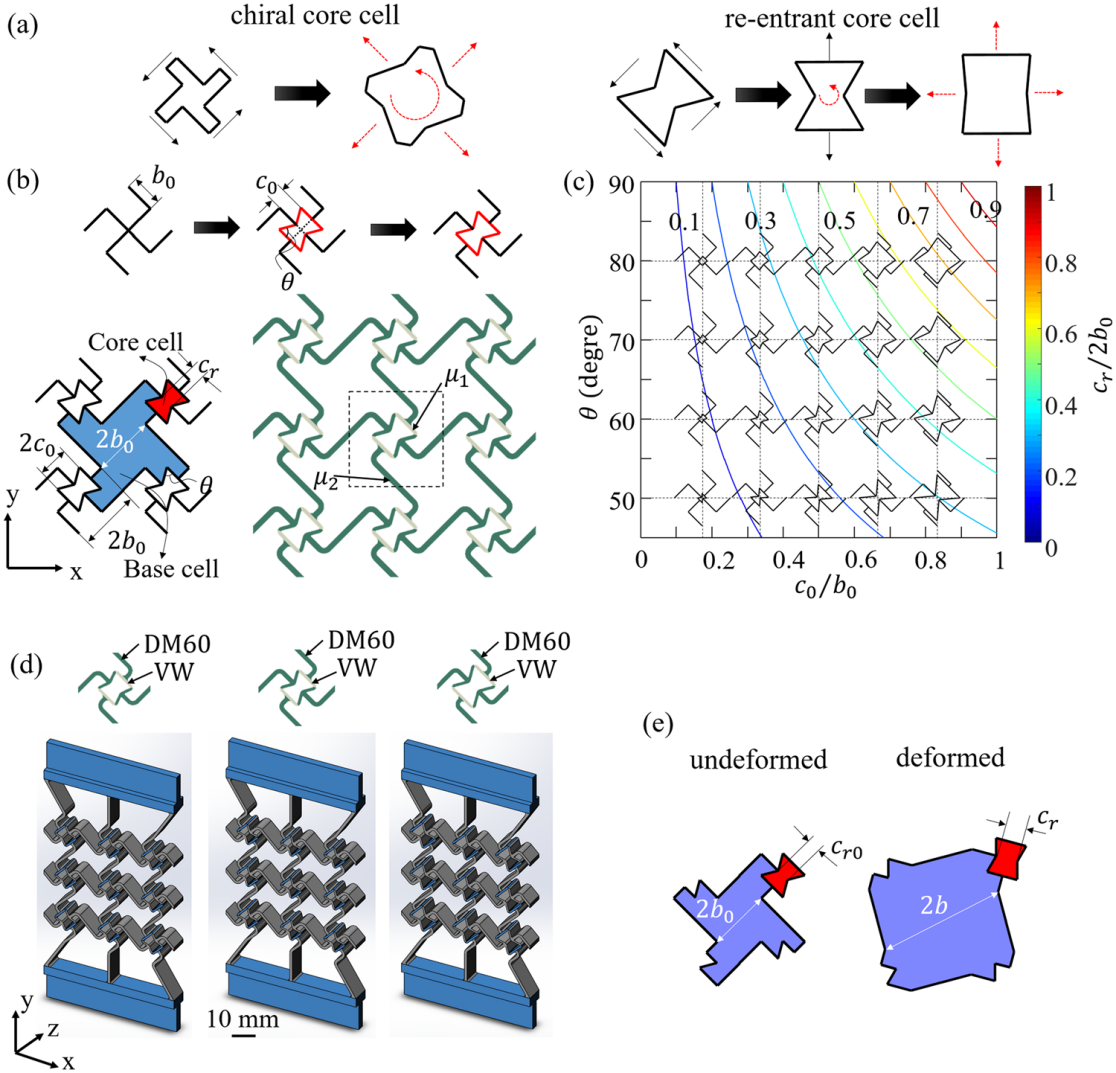
(**a**) The cell opening mechanisms of chiral core cell and re-entrant core cell; (**b**) the concept of generating a new chiral cell with re-entrant core cell (top) and the two types of cells in the new periodic chiral metamaterial (bottom); (**c**) the relation between cell size ratio $\,{c}_{r}/2{b}_{0},$ and the cell size ratio ${c}_{0}/{b}_{0}$ and the re-entrant angle *θ*; (d) the three specimens of the new chiral metamaterial with ${c}_{0}/{b}_{0}=0.5,\,0.5\,{\rm{a}}{\rm{n}}{\rm{d}}\,0.67$ and re-entrant angle $\theta ={70}^{\circ },\,{60}^{\circ },{\rm{a}}{\rm{n}}{\rm{d}}\,{60}^{\circ }$ for Specimens 1, 2 and 3, respectively; (**e**) the schematic drawing showing the change in size of each cell during deformation.

As explored recently^56^, by introducing an auxetic core cell to a chiral base cell, a sequential cell opening mechanism can be achieved. Based on the two basic deformation mechanisms for auxetic effects, there are actually two options for the core cell geometry: chiral geometry and re-entrant geometry (Fig. 1a). Due to the different geometry, the deformation mechanisms of the two different core cells are quite different. Conceptually, as shown in Fig. 1a, for the chiral core cell, cell rotation and cell opening are always coupled, although during the initial cell rotation, the cell opening can be small^56^; while for the re-entrant core cell, cell rotation and cell opening are independent, which means when the re-entrant core cell rotates, it barely opens, and it opens only when it is stretched. Thus, it is expected that compared with the first design option with chiral core cell^56^, the latter one with re-entrant core cell can have a much larger overall strain to trigger the core cell opening.

To explore the second design option, new chiral mechanical metamaterials with re-entrant core cells were designed (Fig. 1b). The missing-rib type of chiral cell with rib-length ${b}_{0}$ was used as the starting geometry (Fig. 1b, top). A bowtie sharped re-entrant core cell was added at the center of the chiral cell. Then the four ribs in the center were disconnected from the cell center O, and formed an open core cell. Thus a unit cell of a new periodic chiral metamaterial was generated.

In this way, the new hybrid mechanical metamaterial has two types of cells: (1) base chiral cells with major size $2{b}_{0}$ (highlighted in blue in Fig. 1b, left), and (2) re-entrant core cells (highlighted in red in Fig. 1b, left) with major size $2{c}_{0}$. The cell size ratio ${c}_{0}/{b}_{0}$ between the two types of cells is an important non-dimensional parameter to determine the geometry of the new cell. Since ${c}_{0}$ is always less than $\,{b}_{0}$, ${c}_{0}/{b}_{0}$ can vary between 0 to 1. Other geometric parameters include the distance ${c}_{r}$ between the two re-entrant tips in the core cell, and the re-entrant angle $\theta$. The initial ratio ${c}_{r}/2{b}_{0}$ between the core cells and base cells is related to the initial length ratio ${c}_{0}/{b}_{0}$ and re-entrant angle $\theta$ via:

1 $${c}_{r}/2{b}_{0}={c}_{0}/{b}_{0}(1-\,\cos \,\theta )$$

According to Eq. (1), the size ratio ${c}_{r}/2{b}_{0}$ between the core cells and the base cells can be tailored by the cell size ratio $\,{c}_{0}/{b}_{0}$ and the re-entrant angle $\theta$, as shown in Fig. 1c. When ${c}_{0}/{b}_{0}$ and $\theta$ increase, ${c}_{r}/2{b}_{0}$ will increase. ${c}_{r}/2{b}_{0}$ can be tuned between 0 to 1 as well. Thus, as shown in Fig. 1c, the geometry of the new hybrid cell is determined by any two of the three non-dimensional geometric parameters,$\,{c}_{0}/{b}_{0}$, ${c}_{r}/2{b}_{0}$, and $\theta$.

It is known that the bending of the connecting ribs (highlighted as grey in Fig. 1b) can significantly reduce the re-entrant effect of the core cells. So to achieve better auxetic performance, a multi-material design strategy is used to reduce the bending of the connecting ribs and therefore to achieve better auxetic effects for the core cell. In the design, stiffer material is used for the connecting ribs in the core cells, as shown in Fig. 1b (right), in which the rib material has a larger shear modulus ${\mu }_{1}$, and the major material (dark green color in Fig. 1b) has a smaller shear modulus ${\mu }_{2}$. An alternative single-material design is provided in the supporting material S1.

To explore the influences of the core size (determined by ${c}_{0}/{b}_{0})\,\,$and the re-entrant angle (determined by $\theta )\,\,$ on the deformation mechanisms of the new chiral metamaterial, three specimens were designed as shown in Fig. 1d. The dimensions of the major part of all three specimens are 75 mm × 75 mm × 20 mm along *x*, *y* and *z* directions, respectively. The in-plane (*x*-*y* plane) thickness of the ribs is 1.5 mm, the cell size ratio ${c}_{0}/{b}_{0}$ are 0.50, 0.50 and 0.67, respectively, and the re-entrant angle $\theta$ are 70, 60 and 60 degree, respectively. The size of the base cells $\,({b}_{0}=5.30\,mm)$ is the same for all three specimens. To reduce the boundary effects, straight linkages were added between the top and bottom boundaries of the specimens and the shoulders. Thus, the only difference between Specimens 1 and 2 is the angle $\theta$, and the only difference between Specimens 2 and 3 is the cell size ratio ${c}_{0}/{b}_{0}$.

The three designs were then fabricated via a multi-material 3D printer (Objet Connex 260). All three specimens were printed with the same two materials: the major material DM9760 (with shear modulus ~0.92 MPa) and the connecting rib material VeroWhite (with Young’s modulus ~2 GPa, Poisson’s ratio ~0.35, shear modulus ~740.7 MPa); Therefore, the only difference between Specimens 1 and 2 is the re-entrant angle $\,\theta$. The only difference between Specimens 2 and 3 is the cell size ratio $\,{c}_{0}/{b}_{0}$.

To evaluate the cell opening mechanisms of both the core cell and the base cell during deformation, a nondimensionalized cell opening factor (*CoF*) is introduced as $Co{F}_{c}$ and $Co{F}_{b}$, respectively:

2 $$Co{F}_{c}=\frac{{c}_{r}-{c}_{r0}}{{c}_{r0}},$$

3 $$Co{F}_{b}=\frac{b-{b}_{0}}{{b}_{0}},$$

where, ${c}_{r0}$ and $2{b}_{0}$ are the initial sizes of the core cell and the base cell, respectively, and ${c}_{r}$ and 2*b* are the sizes of the corresponding cells under deformed configuration, as shown in Fig. 1e.

### Experiments vs. FE simulations

**Figure 2:**
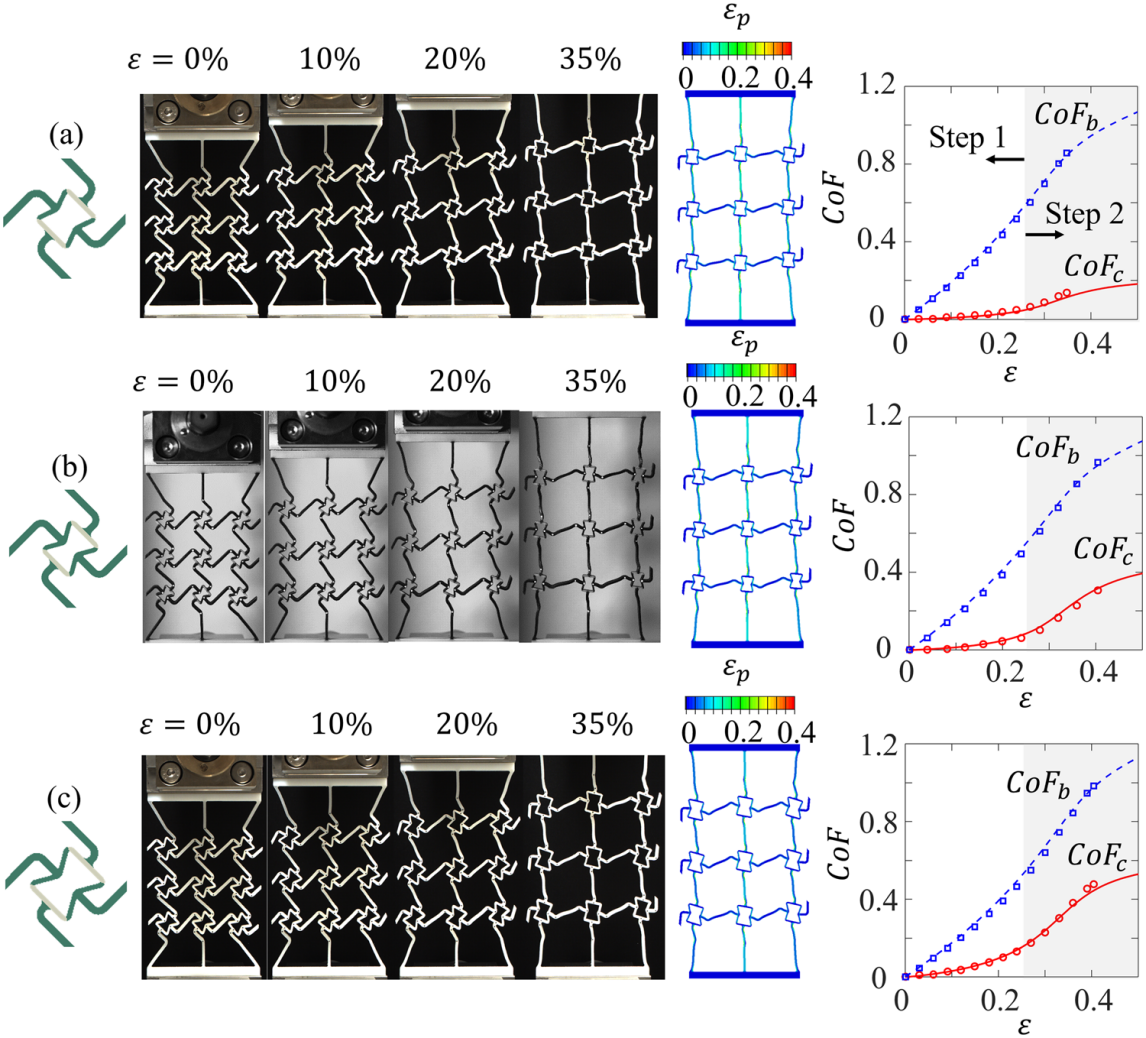
Experimental and FE results of (**a**) Specimen1: snap-shots of the deformed configurations at different overall strains (left) and the curves of *CoF*s vs. overall strain (right); (**b**) Specimen 2: snap-shots of the deformed configurations at different overall strains (left) and the curves of *CoF*s vs. overall strain (right); and (**c**) Specimen 3: snap-shots of the deformed configurations at different overall strains (left) and the curves of *CoF*s vs. overall strain (right).

Uni-axial tension experiments (Videos are provided in the supporting material. Videos 1, 2, and 3 are for Specimens 1, 2 and 3, respectively) and FE simulations were performed for all three specimens (details are provided in the section of Methods). The experimental and FE results of the three specimens are shown in Fig. 2a–c, respectively. All specimens showed auxetic effects so that the horizontal dimensions of all specimens increase under the vertical tension. The deformed configuration and the FE contours of the maximum principal in-plane strain for three specimens are also shown in Fig. 2 to compare with experimental results. It can be seen that at ~35% overall tensile strain, the deformed configurations from the FE simulations are very similar to those of the corresponding experiments.

The FE and experimental results of the *CoF*s of the core cell and the base cell for each specimen are plotted as functions of the overall tensile strain *ε* in Fig. 2a–c, respectively. It can be seen that for all three specimens (Fig. 2a–c, middle), initially, $Co{F}_{b}$ increases more rapidly than $Co{F}_{c}$, as indicates that the base cells open first; while, $Co{F}_{c}$ is almost zero and barely changes at the beginning. When the overall strain increases beyond ~20%, $Co{F}_{c}$ starts to increase rapidly, as indicates the core cells start to open. The experimental results show a sequential cell-opening mechanism with base chiral cell opens much faster than the re-entrant core cell. Although, the opening rate of the base cells is quite similar for different geometries, the opening rate of the core cells can be tuned widely by tailoring the geometry. Also, it was observed that for all three specimens, the core cells and base cells rotate in opposite directions: the core cells rotate counter-clockwise (positive) and the base cells rotate clockwise (negative).

**Figure 3:**
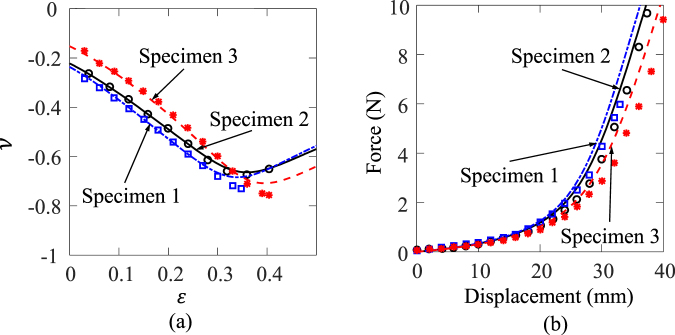
Experimental and FE results (lines represent FE results and symbols represent experimental results) of (**a**) Poisson’s ratio vs. overall strain for all three specimens, and (**b**) load-displacement curves of all three specimens.

The load-displacement curves of the three specimens are shown in Fig. 3a. It can be seen that for Specimens 1, 2 and 3, the Poisson’s ratios initially are ~−0.22, ~−0.21 and ~−0.15, respectively, and then decrease during deformation. When the wavy ribs are all stretched up along the loading direction, the Poisson’s ratios reach a valley (with Poisson’s ratio about −0.8) and then start to increase. The overall strain for Specimens 1, 2 and 3 reaching the minimum Poisson’s ratios are ~0.34, ~0.36 and 0.40, respectively.

The load-displacement curves of the three specimens are shown in Fig. 3b. It can be seen that for all three specimens, the overall load-displacement behaviour is hyperelastic with a smaller stiffness at the beginning and then dramatically harden after a certain strain. The hardening is due to the rib stretching after the straightening of the curved ribs. Specimens 1 is the stiffest, followed by the Specimen 2 and Specimen 3 is the softest. The FE prediction and experimental results are consistent.

### More design options

**Figure 4:**
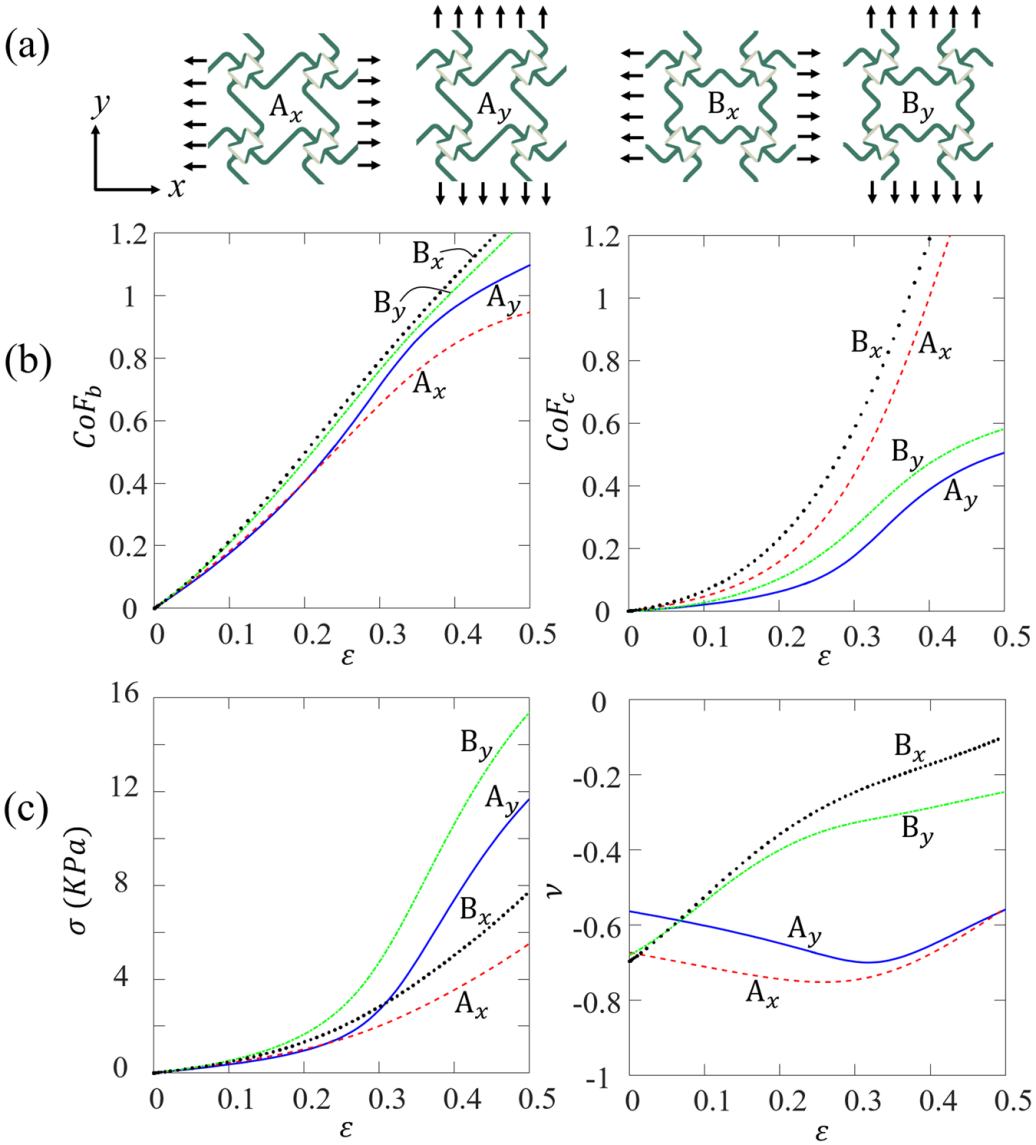
(**a**) The geometries of the four more design options; (**b**) the curves of ${Co}{{F}}_{{b}}$ (left) and ${Co}{{F}}_{{c}}$ (right) vs. overall strain for the four cases; (**c**) the stress-strain curves (left) and the curves of Poisson’s ratio vs. overall strain (right) for all four cases.

For the current hybrid design, the handedness of the base cells is the same, and the re-entrant angles of the core cells are connecting to the passive ribs. By alternating the handedness of the base cells, and the orientation of the core cells, more design options can be developed. FE models of four design options are setup, as shown in Fig. 4a: *A*_*x*_ represents the design with the base cells having the same handedness and the re-entrant angles of the core cells connecting to the active ribs (this happens to be the original design loading in *x* direction); *A*_*y*_ represents the design with the base cells having the same handedness and the re-entrant angles of the core cells connecting to the passive ribs (this happens to be the original design loading in *y* direction); *B*_*x*_ represents the design with base cells having alternating handedness and the re-entrant angles of the core cells connecting to the active ribs; and *B*_*y*_ represents the design with the base cells having alternating handedness and the re-entrant angles of the core cells connecting to the passive ribs.

For all FE simulations, periodic boundary conditions were applied on all four edges of the unit cells. For all four cases, the cell opening factors *CoF*_*b*_ and *CoF*_*c*_ of base cells and core cells are output and compared in Fig. 4b (left and right), respectively. As shown in Fig. 4b, for all four cases, the base cells open faster than the core cells, and the cell opening rates for the base cells are almost the same. While the cell opening rates of the design *B*_*x*_ is the largest, followed by *A*_*x*_, *B*_*y*_ and *A*_*y*_. The effective stress-strain curves (Fig. 4c left) show that the stress-strain relationships of the four cases are very similar at small deformation (less than 10% overall strain) and all curves harden at large deformation. But the hardening slopes are quite different: *B*_*y*_ has the largest hardening slope, followed by *A*_*y*_, *B*_*x*_ and *A*_*x*_. As for the Poisson’s ratio, for the *A*_*x*_ and *A*_*y*_ cases, the Poisson’s ratios first slightly decrease and then increase when the overall strain reaches ~30%; while for the *B*_*x*_ and *B*_*y*_ cases, the Poisson’s ratios increase first and then slightly slow down when the overall strain reaches ~20%.

Among the four design options, *A*_*y*_ has the largest difference between the cell-opening rates of the base cells and core cells, indicating the largest sequential cell opening steps. Also, the Poisson’s ratio of *A*_*y*_ is close to a constant under finite deformation, indicating stable auxetic effects. Therefore, for the parametric studies in the following sections, we focus on design *A*_*y*_.

### Parametric study on the Poisson’s ratio and the effective stiffness

To further explore the mechanical behaviour of the new designs in a large design space, a thorough parametric study was performed via FE simulations. In the parametric study, we focused on the two most important design parameters: the cell size ratio ${c}_{0}/{b}_{0}$, and the re-entrant angle $\theta$. The influences of each parameter on the initial Poisson’s ratio and the overall effective stiffness were quantified. To exclude the boundary effects and get the intrinsic mechanical properties of the material, periodic boundary conditions were used in all FE simulations. Uniaxial tensile loads were applied at the boundaries.

Specifically, in all FE models, unit cell size ${b}_{0}$, and the shear modulus ${\mu }_{1}$ and ${\mu }_{2}$ were kept the same (${b}_{0}$= 5.30 mm,$\,{\mu }_{1}=260$ MPa, ${\mu }_{2}=\,$0.26 MPa), and by varying ${c}_{0}$ from 1.50 mm to 3.92 mm with seven different values, ${c}_{0}/{b}_{0}$ varied as $0.28,\,0.33,\,0.42,\,0.50,\,0.58,\,0.67,\,\mathrm{and}\,0.74$, respectively. The re-entrant angle $\theta$ varied as seven different values: 50, 55, 60, 65, 70, 75, and 80 degrees. Thus, total 49 different FE simulations were performed for this parametric study.

**Figure 5:**
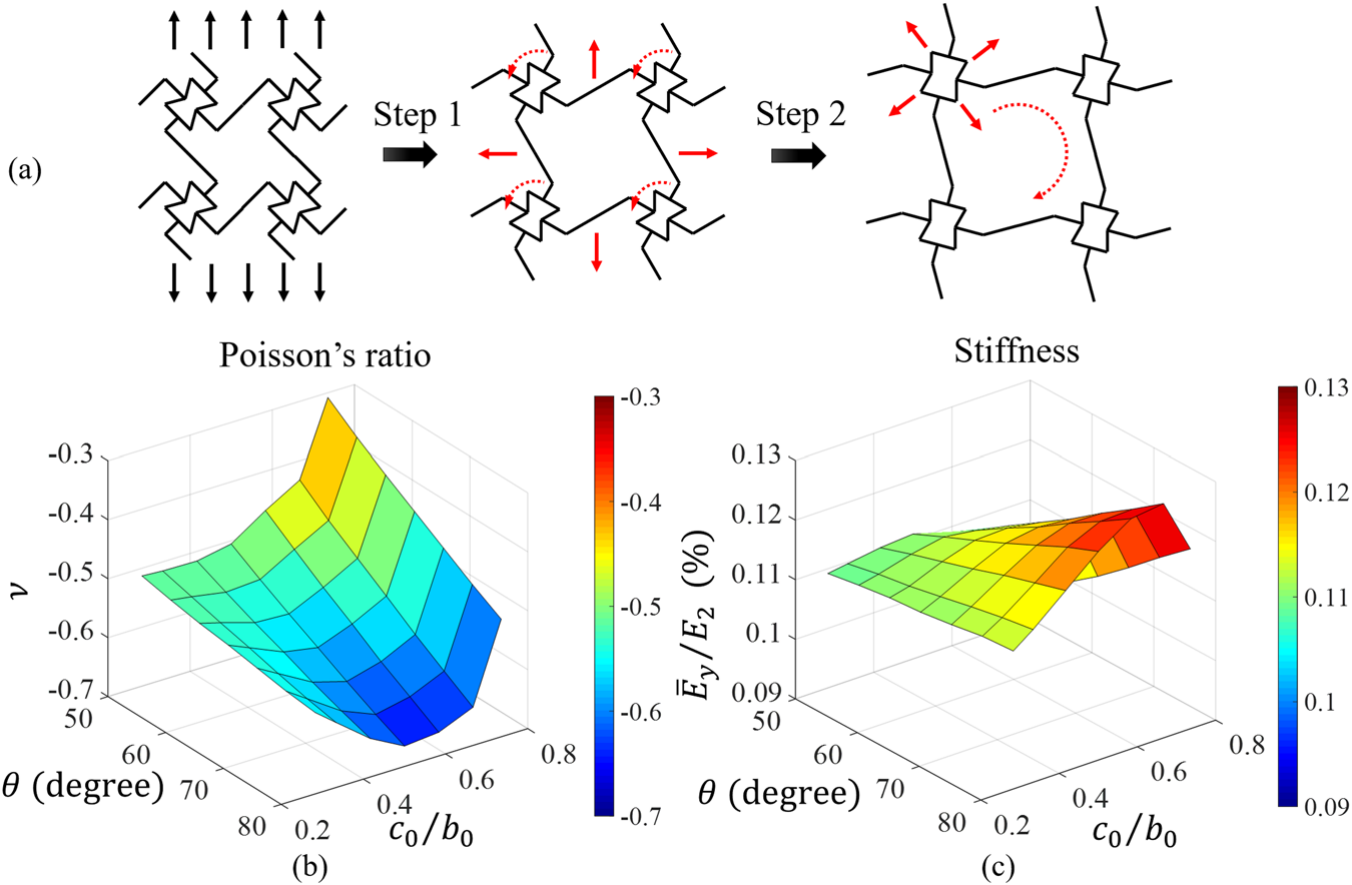
(**a**) The schematics of sequential cell opening, (**b**) the Poisson’s ratios for all FE models, and (**c**) the non-dimensionalized effective stiffness for all FE models.

The FE results of the initial Poisson’s ratio are plotted as functions of cell size ratio ${c}_{0}/{b}_{0}$ and re-entrant angle $\theta$ in Fig. 5a. It can be seen that for all various ${c}_{0}/{b}_{0}$, when the re-entrant angle $\theta$ increases, the Poisson’s ratio $\nu$ will decrease monotonically. However, when ${c}_{0}/{b}_{0}$ increases, the Poisson’s ratio will decrease first and then increase after reaching a valley. For example, when $\theta =70^\circ$, when ${c}_{0}/{b}_{0}$ increases to 0.50, the Poisson’s ratio decreases from −0.55 to −0.65 and then increases when ${c}_{0}/{b}_{0}$ increases beyond 0.50. For parameters chosen, the Poisson’s ratio can be tuned in a large range, from ~−0.3 to ~−0.65.

The FE results of the initial effective stiffnesses in loading direction (*y* direction) are nondimensionalized by the Young’s modulus *E*_2_ of the major material and are plotted as functions of cell size ratio ${c}_{0}/{b}_{0}$ and re-entrant angle *θ* in Fig. 5b. It can be seen that when the re-entrant angle *θ* increases, the stiffness always increases. However, when ${c}_{0}/{b}_{0}$ increases, the stiffness will increase first and then decrease after reaching a peak.

### Systematic quantification on cell opening and particle release mechanisms

**Figure 6:**
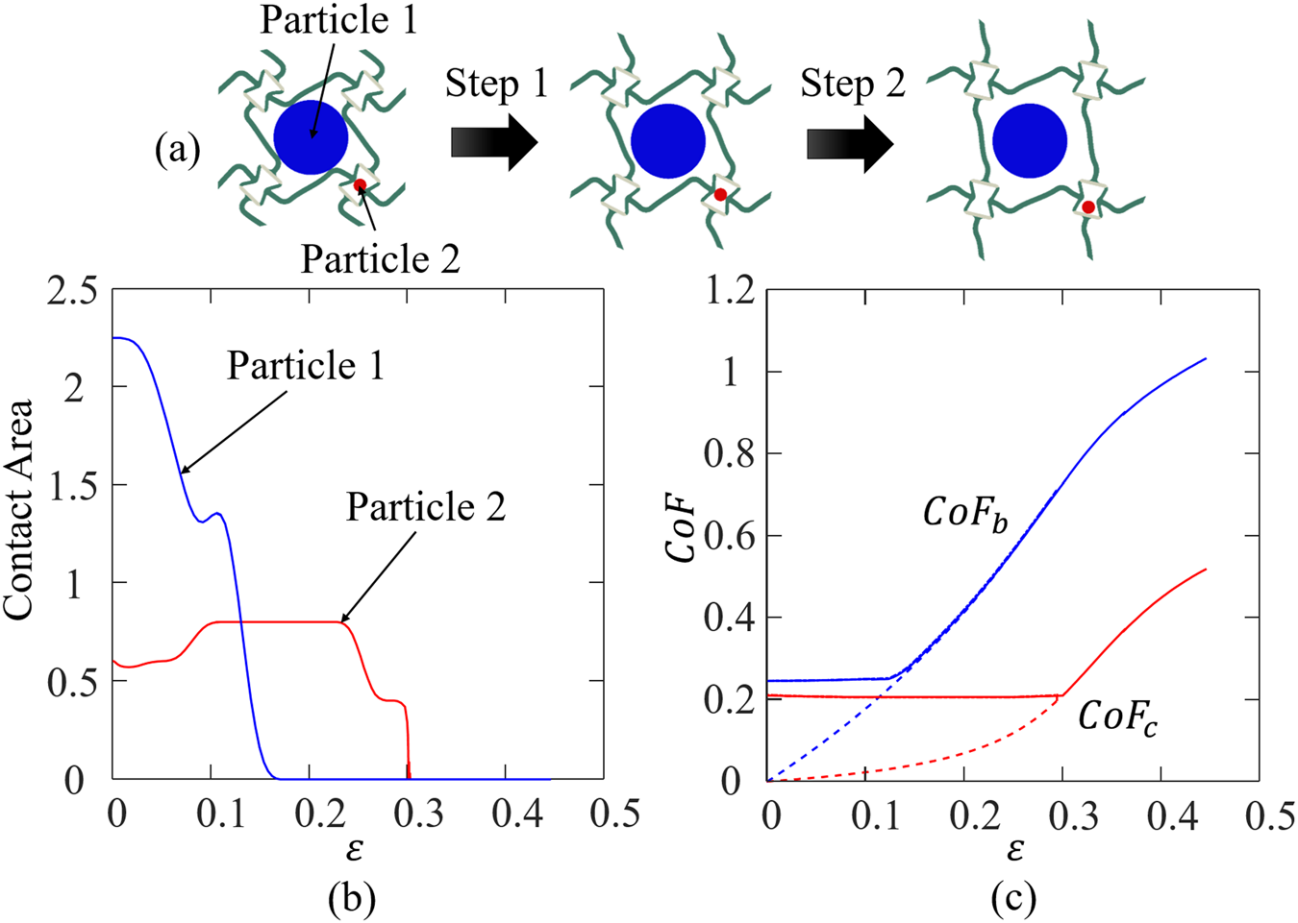
(**a**) The schematics of subsequential particle release, (**b**) FE results of the contact area between particles and the cell walls vs. overall strain, and (**c**) the *CoF*s vs. overall strain (dash line: without particles, and solid line: with particles).

The unique sequential cell opening mechanisms can be used to design sequential particle release mechanisms. A demo for the particle release (Specimen 2) under uniaxial tension is shown in the supporting material (Video 4). FE simulations were performed to study the particle release mechanism (details are provided in section Methods). The FE results are shown in Fig. 6.

Figure 6a shows the three stages of deformation (from left to right) and therefore the sequential particle release mechanism: (i) initial stage, both particles are hold, (ii) big particle is released from the base chiral cell while the small particle is still hold in the re-entrant core cell, and (iii) both big and small particles are released. The three stages are quantified by the evolution of contact area between each particle and the cell wall. The contact areas are output as functions of the overall strain in Fig. 6b, in which the blue curve represents the contact area of the big particle, and the red curve represents that of the small one. Figure 6b shows that the contact area of the big particle held in the base cell becomes zero when the overall strain reaches ~0.16, indicating the release of the big particle; when the overall strain reaches ~0.3, the contact area of the small particle held in the re-entrant core cell becomes zero, indicating the release of the small particle.

The sequential particle release mechanism is due to the sequential cell-opening of the hybrid design. The evolution of *CoFs* are output from FE simulations and are plotted in Fig. 6c (dash line represents the case without particle and the solid line represents the case with particles embedded). For the case with particles, the *CoF*s of both cells start at about 0.2 and keep unchanged until the particle release occurs. After particles are released, the *CoF*s for the cases with and without particles are identical.

**Figure 7:**
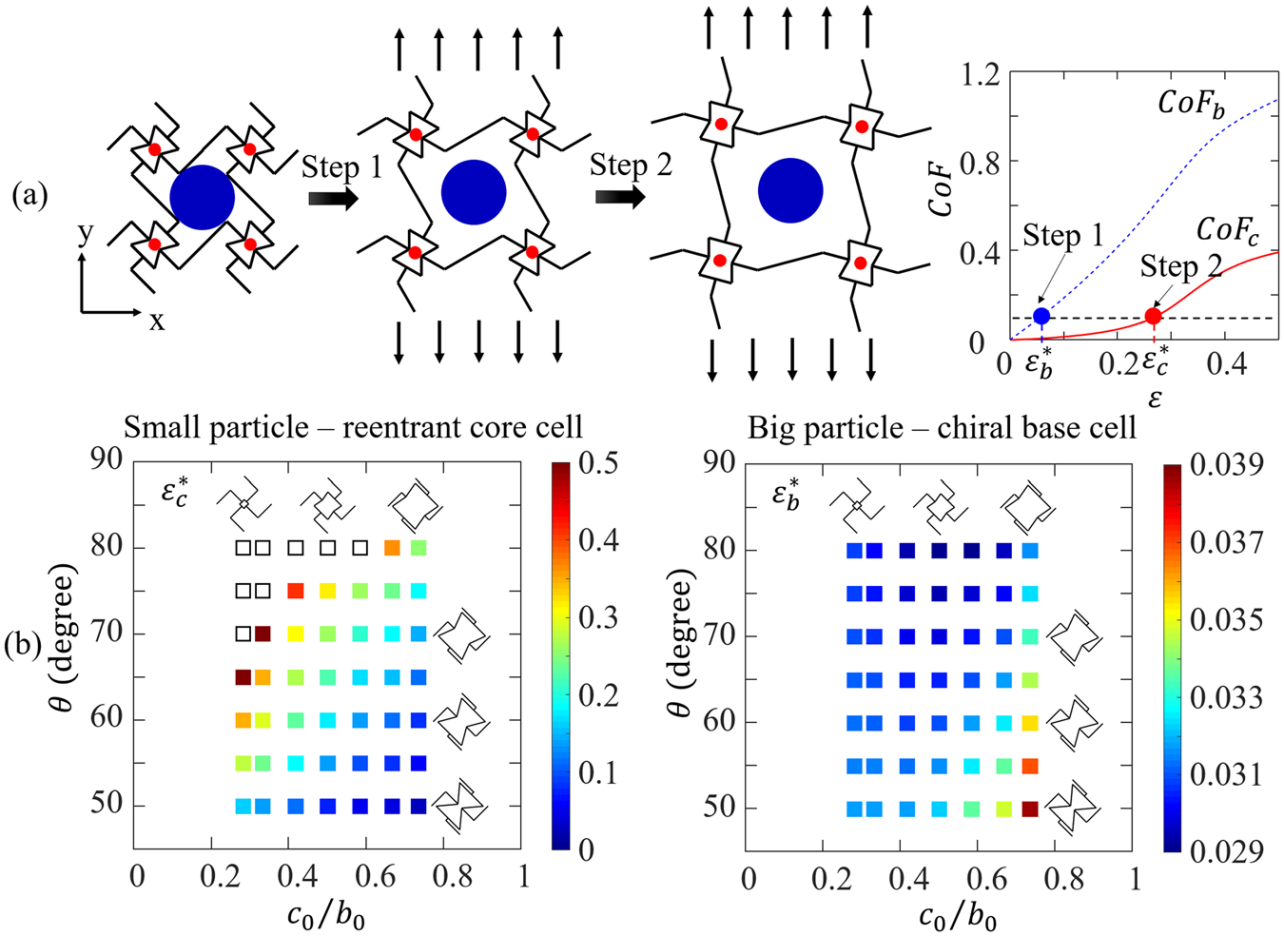
The sequential particle release mechanisms, (**a**) the schematics of the mechanism of bigger particle releasing first, followed by the release of smaller particle, (**b**) the overall strain ${\varepsilon }_{c}^{\ast }$ (left) and ${\varepsilon }_{b}^{\ast }$ (right) for releasing the smaller and bigger particles as a function of ${{c}}_{0}/{{b}}_{0}$, and ${\theta }$.

To systematically explore the particle release mechanisms, 49 FE simulations with cell parameters the same as before were performed. The particles were assumed to be 10% larger than the corresponding initial cell size. The overall strains for releasing the base cell and core cell are defined as ${\varepsilon }_{b}^{\ast }$ and ${\varepsilon }_{c}^{\ast }$, respectively. Thus from the FE simulations, ${\varepsilon }_{b}^{\ast }$ and ${\varepsilon }_{c}^{\ast }$, are the strains corresponding to *CoF*_*b*_ and *CoF*_*c*_ equaling to 0.1, respectively, as shown in Fig. 7a (right). ${\varepsilon }_{b}^{\ast }$ and ${\varepsilon }_{c}^{\ast }$ of the forty-nine FE models were plotted as functions of cell size ratio ${c}_{0}/{b}_{0}\,\,$and re-entrant angle $\theta$ in Fig. 7b.

Figure 7b shows that when ${c}_{0}/{b}_{0}$ decreases and/or $\theta$ increases, ${\varepsilon }_{c}^{\ast }$ will increase. While for ${\varepsilon }_{b}^{\ast }$, when ${c}_{0}/{b}_{0}$ increases, ${\varepsilon }_{b}^{\ast }$ will first slightly decrease and then will increase; and when $\theta$ decreases, ${\varepsilon }_{b}^{\ast }$ will increase. For all parameters chosen, ${\varepsilon }_{b}^{\ast }$ is smaller than ${\varepsilon }_{c}^{\ast }$, indicating the big particle will release earlier than the small particle. The extreme cases are marked by hollow symbols on Fig. 7b, for which ${\varepsilon }_{c}^{\ast }$ will be very large and the small particle will not be released until very large overall strain. Those extreme cases are beyond our interests in practical design.

### Comparison between the two designs with re-entrant cores and chiral cores

**Figure 8:**
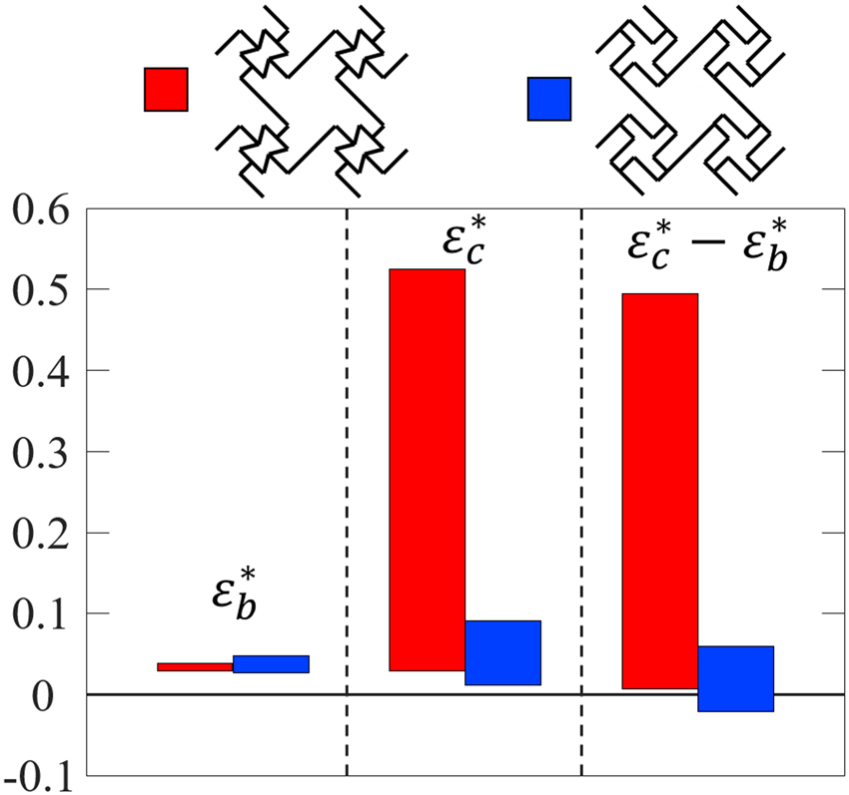
Direct comparison between the designs with chiral core cells and the current design with re-entrant core cells, the ranges of ${\varepsilon }_{b}^{\ast }$, ${\varepsilon }_{c}^{\ast }$ and ${\varepsilon }_{c}^{\ast }\,\mbox{--}\,{\varepsilon }_{b}^{\ast }$ for both designs (the data of the design with chiral core cells are from ref.^56^).

Due to the two different deformation mechanisms of the chiral core cell^56^ and re-entrant core cell (Fig. 1a), although both the current design and the one explored in ref.^56^ have sequential cell opening mechanisms, the cell opening behaviours of them are quite different. The ranges of the overall cell-opening strains of the two designs are directly compared in Fig. 8. Those strains include the overall strain for base cell opening, ${\varepsilon }_{b}^{\ast }$, the overall strain for core cell opening, ${\varepsilon }_{c}^{\ast }$, and the difference between them ${\varepsilon }_{c}^{\ast }-{\varepsilon }_{b}^{\ast }$. The sign of ${\varepsilon }_{c}^{\ast }-{\varepsilon }_{b}^{\ast }$ represents the order of the cell opening: if ${\varepsilon }_{c}^{\ast }-{\varepsilon }_{b}^{\ast }$ > 0, base cells open first, followed by the core cells; if ${\varepsilon }_{c}^{\ast }-{\varepsilon }_{b}^{\ast }$ < 0, core cells open first, followed by the base cells, and if ${\varepsilon }_{c}^{\ast }-{\varepsilon }_{b}^{\ast }$ = 0, both the core cells and base cells open simultaneously.

Since both designs have chiral base cells, for base cell opening, the ranges of the overall strain ${\varepsilon }_{b}^{\ast }$ of the two designs are similar and are both relatively small, as shown in Fig. 8: ${\varepsilon }_{b}^{\ast }$ of the design with chiral core cells^56^ can be tailored from 2.72% to 4.82%, while ${\varepsilon }_{b}^{\ast }$ of the design with re-entrant core cells can be tuned from 2.94% to 3.86%. However, for the core cell opening, the ranges of the overall strain $\,{\varepsilon }_{c}^{\ast }$ of the two designs are very different: ${\varepsilon }_{c}^{\ast }\,\,$of the design with chiral core cells^56^ can be tuned from 1.22% to 9.07%, while ${\varepsilon }_{c}^{\ast }$ of the design with re-entrant core cells can be tuned in a much larger range of 2.91% to 52.6%. This indicate that for the sequential cell opening, the working strain for the design with chiral core cells in ref.^56^ is within 10%; while the working strain for the current design with re-entrant core cells can reach ~50%.

Also, the range of ${\varepsilon }_{c}^{\ast }-{\varepsilon }_{b}^{\ast }$ of the two designs are quite different. For the design with chiral core cells^56^, ${\varepsilon }_{c}^{\ast }-{\varepsilon }_{b}^{\ast }$ can be tuned from~−2.10% to 5.98%, indicating that based on different design needs, by tailoring the geometry, the order of cell opening can be flipped. While for the design with re-entrant core cells, ${\varepsilon }_{c}^{\ast }-{\varepsilon }_{b}^{\ast }$ can be tuned from 0.68% to 49.45%, which is a much larger range on the positive side. Thus, compared with the design with chiral core cells^56^, for the current design, the base cells always open first, and the core cell can open under a much larger overall strain.

In addition, for the design with chiral core cells^56^, softer hinges are needed to achieve the desired sequential cell opening mechanism. While, for the current design with re-entrant core cells, the softer hinges are not needed, the current design can be printed as single material (details of the alternative single-material design are provided in the supporting material S1).

## Conclusions and Discussions

In summary, based on the concept of chirality-induced rotation, new hybrid auxetic metamaterials were designed by introducing re-entrant core cells to the center of a basic chiral cell. The new designs were proved to have subsequential cell-opening mechanisms under a very large range of overall strain, 2.91% to 52.6%. from mechanical experiments on 3D printed prototypes and systematic FE simulations, Two non-dimensional parameters: the cell size ratio ${c}_{0}/{b}_{0}$, and re-entrant angle $\theta$, are identified to be the most important parameters to govern the effective stiffness, the Poisson’s ratio and the cell-opening mechanisms of the new designs. FE simulations of particle release were performed, showing the unique cell-opening mechanism of the new designs can be used for subsequential particle release. The particle release strain for both particles are systematically explored via FE simulations. It was found that when ${c}_{0}/{b}_{0}$ decreases and/or $\theta$ increases, ${\varepsilon }_{c}^{\ast }$ will increase. However, ${\varepsilon }_{b}^{\ast }$ is not sensitive to the geometry.

The new designs have potential wide applications in designing smart mechanical metamaterials which can be responsive to external load and/or environmental conditions such as light, temperature and humidity. The design concepts can be used to develop new material systems, sensors and/or actuators with broad engineering functions such as drug delivery and colour change for camouflage.

## Methods

### Mechanical Experiments

Mechanical experiments of the 3D printed specimens were performed under quasi-static (with overall strain rate ${10}^{-3}$ per second) uniaxial tension. To allow fully curing, all specimens were tested 24 hours after printing under room temperature. The experiments were conducted on a Zwick/Roell material testing machine (ZwickiLine). The shoulders of the specimens were also printed as VeroWhite material and were gripped on the machine. Markers were made on each corner of the cells, and during the experiments, a high resolution camera was used to record the deformed configurations of the specimens at each time instant. By post-processing the images, the displacement history of each marker point was obtained, from which the Poisson’s ratio $\,\nu$, and the cell sizes *b* and *c* were calculated at each overall displacement.

### Numerical simulations

#### Finite element simulations of the experiments

FE simulations of the tensile experiments on the three specimens were performed in ABAQUS/STANDARD V6.13. Four-node 2D plane stress elements (CPS4) were used and the accuracy was verified by mesh refinement study. Since the hard ribs in specimens barely deform during tension, linear elastic material model with Young’s modulus *E* = 2 GPa, the Poisson’s ratio *v* = 0.35, was used (material parameters are measured from standard dogbone tests). For rubbery DM9760, incompressible hyperelastic Mooney-Rivlin model was used. The strain energy density function $W\,$of the Mooney-Rivlin model is $W={C}_{10}({I}_{1}-3)+{C}_{01}({I}_{2}-3)$, where ${I}_{1}$ and ${I}_{2}$ are the first and second stress invariants, respectively. The material parameters were obtained from the standard experiments of both uni-axial tension and compression. For DM9760, ${C}_{01}=0.46{\rm{MPa}}$, ${C}_{10}=0{\rm{MPa}}$, (in the true strain range of ~−0.8 to 0.4). The bottom edge of the FE models was fixed and a prescribed displacement was applied at the top edge.

#### FE simulations of particle release

As one potential application, due to the unique sequential cell opening mechanism, the material can be used to design materials for sequential particle release. FE models for the particle release simulations were developed. For all FE simulations, periodic boundary conditions were used. As shown in Fig. 7a, the geometry of Specimen 2 was chosen for the simulations, one big circular particle (indicated in blue) and one small circular particle (indicated in red) are introduced to the base cell and the core cell, respectively. Both the big and the small particles are ~20% larger than the initial size of the base chiral cells ($2{b}_{0}$) and that of the core cell (${c}_{r}$), respectively. The FE simulations include two major steps: Step 1, to interact and hold the two particles, the hybrid cells were pre-stretched first and then were unloaded after the particles were introduced. Hard contacts were defined between the particles and the cell walls, so that both particles were hold in the cells after unloading. Step 2, a prescribed uni-axial displacement was introduced to the system of cells and particles.

## Electronic supplementary material

S1

S2: Video 1

S2: Video 2

S2: Video 3

S2: Video 4
