# Supplementary material for: 3D Printed Auxetic Mechanical Metamaterial with Chiral Cells and Re-entrant Cores
Source: Sci Rep. 2018 Feb 5;8:2397. doi: 10.1038/s41598-018-20795-2 (PMC5799406; doi:10.1038/s41598-018-20795-2)
Supplement: Supplementary file 1 — S1 [file 41598_2018_20795_MOESM1_ESM.pdf]

Supporting Information

Title: **3D Printed Auxetic Mechanical Metamaterial with Chiral Cells and Re-entrant Cores**

*Yunyao Jiang and Yaning Li**

S1. A single-material design with locally enhanced ribs

During the deformation, the potential bending of the connecting ribs (light grey ones in Fig. S1a (left)) of the re-entrant core cells need to be avoided. Thus, in the main text, these connecting ribs were designed as a stiffer material than other ribs, and multi-material 3D printing is needed. However, an alternative single-material design can also achieve similar mechanical properties and behaviours. The alternative single-material design shown in Fig. S1a (right) is generated by locally thickening the connecting ribs instead of using a stiffer material.

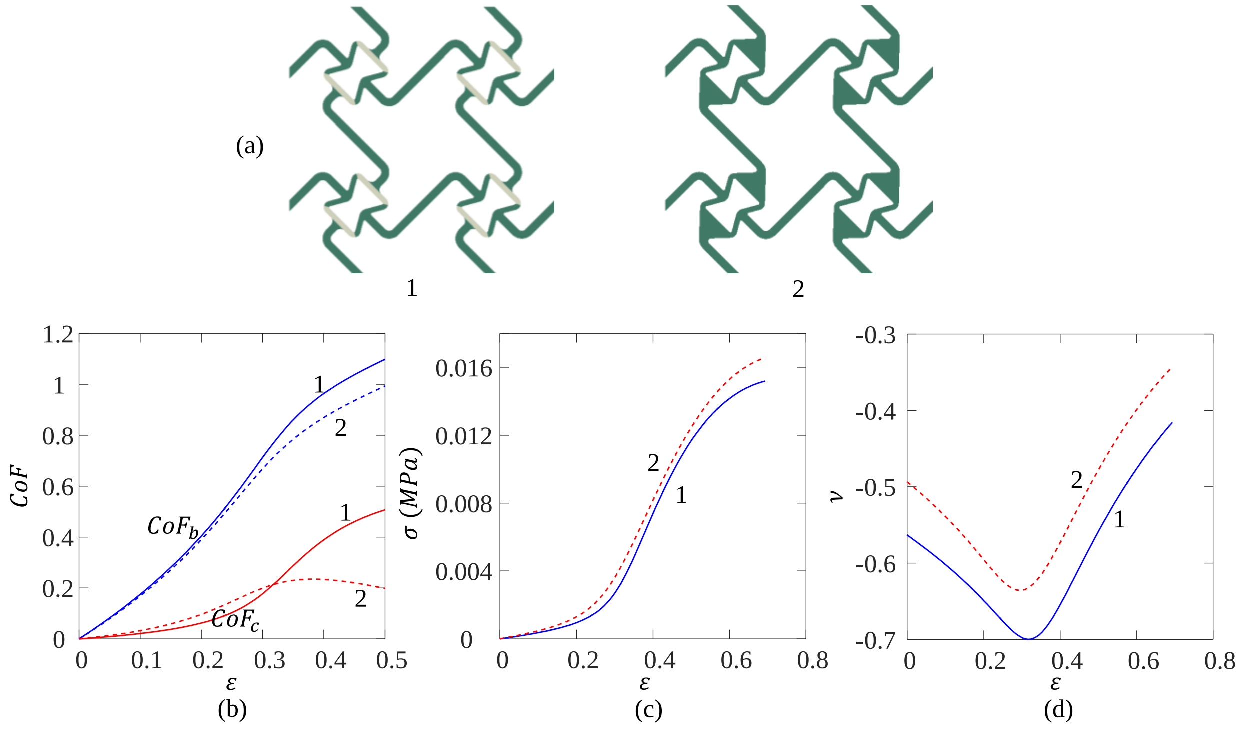

Figure S1. (a) The original multi-material design (left) and the alternative single-material design (right); (b) the comparison of the cell opening factors of the two designs: $CoF_b s$ (blue) and $CoF_c s$

(red) vs. the overall strain; (c) the comparison of the stress-strain curves of the two designs; and (d) the comparison of the Poisson's ratio vs. the overall strain for the two designs.

To verify the alternative single-material design, the FE model of the design is shown in Fig. S1a (right) was developed. The details of the FE simulations are provided in the section of **Methods**. The two designs share the same geometry parameters: $c_0/b_0 = 0.5$ and $\theta = 60°$. The material of the major part (dark green) of the original design is the same as the material in the single-material design.

The FE results of the cell opening factors CoF_b and CoF_c, stress-strain curves, and the Poisson's ratios of the two designs are compared in Figs. S1b, S1c, and S1d, respectively. Fig. S1b shows that when the overall strain is less than 30%, the $CoF_b s$ and $CoF_c s$ of the two designs are very similar, and when the overall strain becomes larger than 30%, the $CoF_b s$ and $CoF_c s$ of the single-material design become lower than those of the original design. Fig. S1c shows that the stress-strain curves of the two designs are very close to each other. Also, for the two designs, the trends of the evolution of the Poisson's ratios are very similar, although the Poisson's ratio of the single-material design is ~0.1 above that of the original design.

In summary, the FE results show that the single-material design has very similar properties and behavior as the original design and therefore can be an alternative design option when multi-material 3D printing is not available.

S2. Videos of mechanical experiments on 3D printed specimens

Video 1. Specimen 1 under quasi-static uni-axial tension (overall strain rate of 10^{-3} per second)

Video 2. Specimen 2 under quasi-static uni-axial tension (overall strain rate of 10^{-3} per second)

Video 3. Specimen 3 under quasi-static uni-axial tension (overall strain rate of 10^{-3} per second)

Video 4. Demo of the particle-release process of Specimen 2 under uniaxial tension
